# The Influence of Examiner Gender on Responses to Tonic Heat Pain Assessments: A Preliminary Investigation

**DOI:** 10.3389/fpain.2021.729860

**Published:** 2021-12-23

**Authors:** Jessica F. McDougall, Nicole G. N. Bailey, Rohan Banga, Lukas D. Linde, John L. K. Kramer

**Affiliations:** ^1^International Collaboration on Repair Discoveries, University of British Columbia, Vancouver, BC, Canada; ^2^Department of Rehabilitation Sciences, Faculty of Medicine, University of British Columbia, Vancouver, BC, Canada; ^3^Department of Anesthesiology, Pharmacology and Therapeutics, Faculty of Medicine, University of British Columbia, Vancouver, BC, Canada; ^4^Djavad Mowafaghian Centre for Brain Health, University of British Columbia, Vancouver, BC, Canada

**Keywords:** quantitative sensory testing, sex differences, gender differences, participant-controlled temperature, thermal pain

## Abstract

**Background:** The influence of examiner gender on pain reporting has been previously explored in both research and clinical settings. However, previous investigations have been limited, with the majority of studies employing single, static assessments of pain (e.g., cold pressor test, verbal pain ratings). The impact of examiner gender on both static and dynamic heat-based pain assessments is currently unknown.

**Methods:** Thirty eight participants (20 females aged 24.1 ± 4.44, and 18 males, aged 24.8 ± 4.54) completed two identical testing sessions, randomized to a male and female examiner in a cross-over design. Pain sensitivity was examined using heat pain thresholds, verbal pain ratings to tonic heat, computerized visual analog scale (CoVAS) rating to tonic heat, and participant-controlled temperature (PCT) heat pain assessments.

**Results:** Female participants reported higher verbal pain to tonic heat with a female examiner compared to male participants, with similar trends for CoVAS responses to tonic heat. Conversely heat pain thresholds and PCT were not significantly influenced by experimenter gender.

**Conclusions:** Overall, verbal ratings were the most impacted by examiner gender, with temperature-based methods such as PCT and pain thresholds showing little to no examiner gender effects. While the gender of the examiner may be an important consideration in the measurement of sex and gender differences in pain research, the choice of pain assessment method may be of similar consequence.

## Introduction

The role of sex and gender on pain has been the source of substantial scientific and public discourse (1–5). In clinical settings, females experience acute and chronic pain with more frequency and to a greater intensity compared to males (2). Experimental studies employing pain sensitivity quantitative sensory testing [QST; a battery of tests which examines noxious and non-noxious somatosensory sensitivity (6)] outcomes (e.g., cold pressor tests, pain pressure thresholds) have provided complimentary support that females may be more sensitive to noxious stimuli than males (2, 4). Heterogeneity among these QST outcomes are commonplace, however, challenging the notion of the aforementioned robust sex or gender-related differences in pain perception (1).

A number of social factors have been proposed to contribute to variation in QST outcomes between experimental pain studies. These include individual and interpersonal factors, as well as environmental factors such as time of day (7, 8). Related to interpersonal factors, the social, gender context of the pain experience appears to influence pain report. Opposing examiner gender effects have been reported, with male participants tending to verbally report significantly less pain in the presence of a female examiner (and vice versa for female participants) (7, 9, 10) [note: gender is used in this regard given that these effects are *social* as opposed to *biological* (5)]. This follows the Gender Context Model of Pain, which suggests men will be less likely to express pain, especially if the examiner is perceived as being threatening to masculine gender roles, whereas women will be more likely to express pain. However, this difference may be dependent on how pain is expressed. Verbal pain report may be more susceptible to these gender differences than non-verbal expressions (11, 12). Indeed, individual factors add complexity. One possible explanation for these reported gender specific examiner effects may be differences in catastrophizing—a negative cognitive-affective response to pain (13). Catastrophizing is associated with increased pain across a variety of pain measures and may be influenced by the presence of others (4, 13). Moreover, sex differences in catastrophizing have been reported, insofar as women tend to catastrophize more than men (4). As such, catastrophizing may also modulate the interaction between sex and social interaction of pain measurements.

A major limitation of previous experimenter/participant gender investigations has been a narrow focus on pain tolerance, measured chiefly by way of the cold pressor test (7). Advances in QST techniques have led to the development of various static and dynamic outcomes, which have been widely employed to investigate sex/gender differences in pain perception (14). Painful thermal dynamic and static QST measures have shown significant differences between male and female participants (4) and may be differently susceptible to experimenter gender influence, and to gender stereotypes. For example, verbal pain ratings of heat pain involve direct verbal communication with experimenters in response to a noxious stimulus, conversely, automated metrics of pain assessment, such as participant controlled temperature (PCT) (15), require less direct communication with examiners. Verbal pain report has been shown to be susceptible to the gender context in which the report occurs (7, 9, 10), however it is not known how susceptible PCT—a non-verbal form of pain expression—is to these gendered influences. It stands to reason that such differences in participant/experimenter interactions within QST assessments may influence the effect of experimenter gender on pain perception. Including both verbal and non-verbal pain reports to both a male and female examiner allows us to tease apart the impact of social context on the apparent sex/gender differences in pain. To our knowledge, no previous studies have explored the influence of experimenter gender on pain outcomes assessed using multiple painful heat QST techniques.

Our aim was to determine the extent to which modern QST heat-pain measures are influenced by the gender of the examiner. To this end, we employed verbal and non-verbal rating and temperature-based (non-verbal) methods of reporting sensitivity to heat pain, with both static and dynamic outcomes. A secondary, exploratory aim explored role of psychosocial factors, specifically the effect of pain catastrophizing on experimenter gender effects on pain outcomes. We expected to see greater gender differences in verbal ratings-based measurements of pain compared to temperature-based measurements, such that males would verbally rate pain as lower in the presence of a female examiner, and females would demonstrate opposite and smaller effect. We anticipate temperature-based methods to show smaller or non-significant effect, as these rely on less direct social interaction during pain reporting.

## Materials and Methods

### Participants

We determined 40 participants (females aged 24.1 ± 4.44, and males, aged 24.8 ± 4.54) would provide a partial eta-squared (${\text{η}}_{\text{p}}^{2}$) = 0.05, with a power of 0.8 and an alpha of 0.5 (calculation completed in G*Power 3.1) (16). This ${\text{η}}_{\text{p}}^{2}$ was estimated from previous studies that have compared the interaction of experimenter and participant gender on pain outcomes (17–19). Exclusion criteria included presence or history of chronic pain (i.e., pain persisting longer than 3 months), determined from a self-reported health history questionnaire. All participants were over 18 years of age and provided informed consent. Participants were recruited from the local university and hospital communities through flier advertisements.

### Experimenters

The experimenters were a cis-female aged 22 and a cis-male aged 19. Both wore a lab coat over jeans and a shirt, and both identified as cis-gendered [i.e., indicated that their gender (man/woman) did not differ from their sex (male/female)]. We did not control for other experimenter characteristics (e.g., height, weight, or race), and these characteristics were not collected from participants. Scripts were created to standardize interactions with the participants, including instructions for all pain tests.

### Procedure

**Figure 1:**
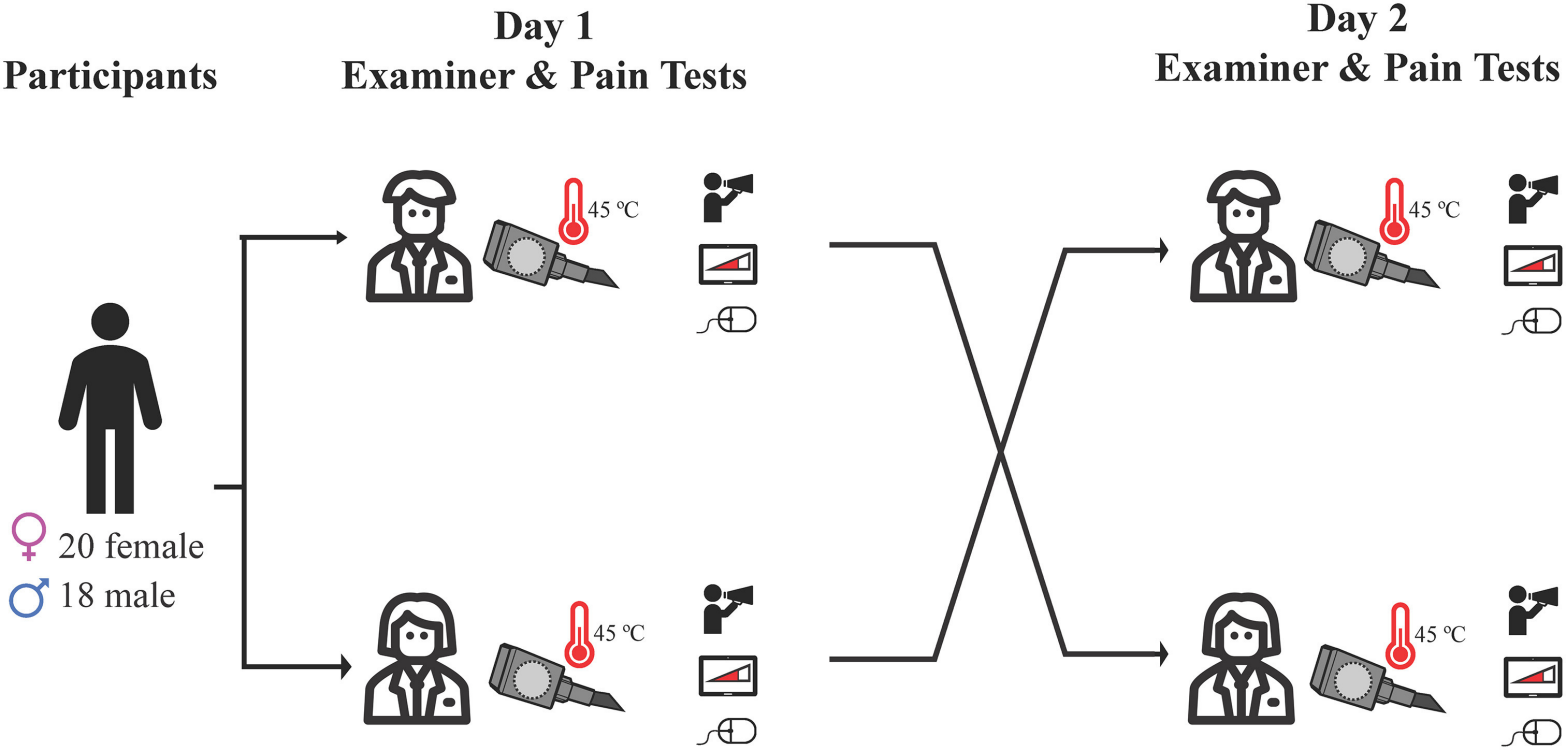
Outline of study protocol. Participants were randomly assigned to a male or female examiner on day 1 in a counterbalanced design, and completed heat pain testing using three different methods: verbal pain rating, computerized visual analog scale, and participant-controlled temperature. Day 2 testing was identical, and was conducted by the opposite gender examiner.

Participants were randomly assigned to a male or female examiner on day 1 in a counterbalanced design, such that half of the participants began with the male examiner, while the other half began with the female examiner (Figure 1). Sessions were at least 24 h apart. Each testing day was designed to be approximately 1-h long. The true nature of the study was withheld from participants, who were led to believe that the purpose was to compare two measures of testing heat pain. Given the blinded nature of our study, the experimenter followed a script that introduced them as the research assistant for the study, with no mention of their gender or the true nature of the study. Experimenters stayed close beside the participant for all tests, standing beside the participant and alternating between watching a computer screen (where the test results were being shown), making an arbitrary note on a clipboard, and glancing at the participant to ensure protocols were being followed. The switch of experimenters was explained to participants as a “scheduling conflict,” and the other experimenter was filling in due to the absence. At the end of day 2, participants were fully debriefed. This involved the experimenter outlining the need for deception and offering participants the opportunity to withdraw their data from the study. All participants were then asked if they suspected or knew the true purpose of the study. All study procedures were approved by the Behavioral Research Ethics Board at the University of British Columbia (approval number H19-00944), and were conducted in accordance with the Declaration of Helsinki (20) involving research on human participants. Our study protocol was not pre-registered, due the required deception of participants (i.e., pre-registering planned statistical comparisons could give away the true nature of the study).

### Heat Pain Measurements

Heat pain thresholds and responses to prolonged heat pain were performed using a calibrated thermode (Medoc Advanced Medical Systems, Ramat Yishai, Israel, CHEPs thermode, 27 mm diameter) applied on the palmar aspect of the forearm. Heat pain thresholds were performed first on the distal 1/3 of a randomly chosen forearm, followed by either PCT or continuous visual analog scale (CoVAS) heat tests performed on the proximal 2/3 of the same forearm—the order of the PCT and CoVAS test presentation was randomized. Prior to the presentation of each heat test, a familiarization test took place to introduce participants to the pain-rating method. A 5-min break separated the three tests (heat pain thresholds, familiarization, and tonic heat test). Following the first tonic heat test, a 10-min break took place. Heat pain thresholds were then performed on the distal 1/3 of the other forearm, followed by the PCT or CoVAS, whichever was randomized to be performed second. Another familiarization test was performed prior to the introduction of the second tonic heat test. Again a 5-min break separated each of the three tests (heat pain thresholds, familiarization, and tonic heat test).

### Heat Pain Thresholds

For heat pain thresholds, the thermode temperature was increased at a rate of 1°C/s from a baseline of 32°C to a maximum of 55°C. Participants were instructed to press a button when the first sensations of pain were perceived (i.e., when the original impression of warmth or heat turned into the feeling of “burning,” “stinging,” “aching,” or “drilling”) (6). Upon button press, the heat thermode returned to the baseline temperature of 32°C at a rate of 70°C/s. Four trials were conducted consecutively with at least 5 s between each trial. The main outcome measure from pain threshold assessments was the average temperature of the initial pain sensations over the four trials.

### Tonic Heat Pain

Participants continuously rated their pain perception throughout a 2-min application of tonic heat (45°C) *via* CoVAS (Medoc Advanced Medical Systems, Ramat Yishai, Israel). The initial temperature of the thermode increased at a rate of 70°C/s, and reached 45°C from a baseline of 32°C, then was maintained at 45°C for 2 min of tonic heat. We chose 45°C for tonic heat pain to maintain similar sensations to the participant-controlled temperature assessment described below (15). At the end of the 2 min, participants also reported their pain verbally to the experimenter (0-10, 0—“no pain at all,” 10—“worst pain imaginable”). Participants were instructed to rate their pain using a slider on the CoVAS machine, which has a visual of a linear increasing graph, indicating no pain on one end and the maximal amount of pain they could tolerate on the opposite end. Participants were asked to rate their pain continuously, moving the slider as desired. The rating was recorded every 20 ms. The average pain rating from the CoVAS readings was recorded as average pain rating to tonic heat.

### Participant Controlled Temperature

For participant controlled temperature (PCT), participants continuously adjusted the temperature of the thermode to maintain their initial perception (15). For example, if at the beginning of the 2-min trial (at 45°C) participants rated the pain as a 4/10, they were instructed to either increase or decrease the temperature in order to maintain the 4/10 sensation over the 2 min. Participants were provided a computer mouse to control temperature, whereby left and right button clicks changed the temperature by ±0.1°C, respectively. Participants were informed that the temperature “may feel as though it is increasing or decreasing,” and were asked to maintain their initial perception by raising or lowering the heat through clicking the mouse. To confirm participants maintained their pain rating throughout the 2 min, each was asked to verbally report their pain at the beginning and end of the protocol. The protocol was identical to that presented by Jutzeler et al. (15). Average temperature across the 2 min of PCT was taken as the primary outcome.

### Familiarization to Heat Pain Assessments

Familiarization trials for both CoVAS and PCT were conducted on a neutral test site. Participants were exposed to 1 min of heat, beginning at a baseline of 40°C. Then, the temperature oscillated by ±2°C at rate of 0.5°C/s. During this time, participants were instructed to rate their pain for CoVAS or to maintain consistent pain sensations via button clicks for PCT. This oscillation in temperature provided participants the opportunity to become accustomed to both heat sensations and the CoVAS and PCT apparatus in response to multiple temperatures. The familiarization trials also helped to reinforce the concept that the temperature in the PCT trials also could be perceived as though it was increasing or decreasing, supporting the blinding of participants to the nature of the PCT trials.

### Questionnaires

At the conclusion of the second day of testing, the pain catastrophizing scale (PCS) questionnaire was administered. The PCS involves the participant rating 13 statements regarding the types of thoughts and feelings that occur when they are in pain from 0 (“not at all”) to 4 (“all the time”). There are three subscales in the PCS; magnifying (three items, “*I become afraid that the pain will get worse*”), rumination (four items, “*I keep thinking about how badly I want the pain to stop*”), and helplessness (six items, “*It's terrible and I think it's never going to get any better”*). Higher PCS scores have been associated with greater levels of pain and pain-focused experiences (21). PCS scores also tend to be higher in females (1).

A demographics questionnaire was also delivered on the first day of testing, asking participants to report their sex, gender, and age. For gender, participants were asked “*What is your gender?”* with options for “female,” “male,” “non-binary/third gender,” “prefer to self describe,” or “prefer not to say.”

### Statistical Analysis

Cohen's d effect sizes were calculated for differences in pain outcomes between male and female participants separately by examiner. This was done to simply model pain outcomes measured by a single examiner of one sex, as would be commonplace in previous studies. The primary outcomes were verbal pain rating following 2 min of tonic heat, average CoVAS rating over 2 min of tonic heat pain, heat pain thresholds, and average temperature over 2 min of PCT assessment. Descriptive statistics were assessed using histograms, box plots, and Q-Q plots to confirm normal distributions of pain outcomes. A preliminary analysis revealed that all pain outcomes were normally distributed (Shapiro-Wilk test range: 0.05-0.29). To formally and comprehensively test our study design, we adopted a repeated measure ANOVAs approach with participant gender as a between-subject variable, and examiner gender as the within-subject variable. Order of testing (i.e., day 1 or day 2) was considered as a covariate to confirm effects were due to the examiner gender and not the repeat-testing nature of the study design. Significant interaction effects were further explored with *post hoc* Bonferroni corrected pairwise comparisons.

Relationships between PCS and pain outcomes were explored using bivariable Pearson correlations, with a Bonferroni correction for multiple comparisons. We examined relationships between pain scores and PCS across both testing sessions as well as explored associations between PCS scores and relative differences in pain scores between testing sessions (i.e., examiners).

## Results

Forty participants were recruited, 38 of which completed both sessions (20 females and 18 males). Missing data from the two subjects was due to technical issues with the heat stimulator—they were unable to complete either day of testing. All other subjects completed both experimental sessions. No subjects withdrew their data after debriefing. Upon debrief, all participants confirmed no knowledge of the true purpose of the study. All participants identified as cis-gendered.

### Rating Based Methods

**Figure 2:**
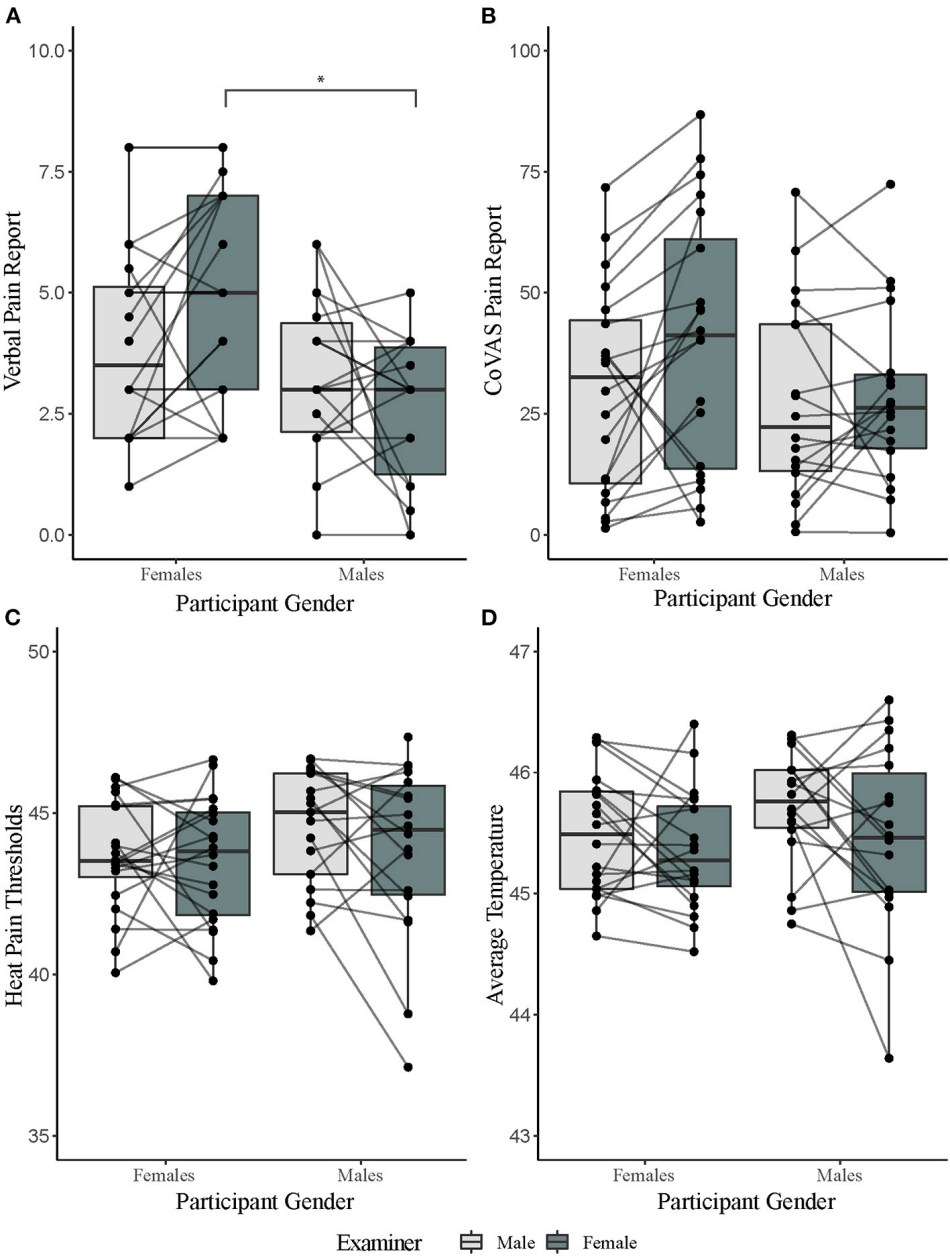
Results of pain tests separated by participant and examiner gender. **(A)** Verbal pain reports for a 2-min tonic heat test separated by gender. **(B)** computerized visual analog scale (CoVAS) reports for a 2-min tonic heat test separated by gender. **(C)** Average heat pain threshold reports separated by gender. **(D)** PCT reports (as calculated by average temperature over a for a 2-min tonic heat test) separated by gender. ^*^denotes significance level of *p* < 0.05 from Bonferroni corrected *post hoc* analysis.

There was a significant main effect of participant gender on verbal pain rating to tonic heat [*F*(_1,36)_ = 5.77, *p* = 0.02, ${\text{η}}_{\text{p}}^{2}$ = 0.14]. This suggests that female participants verbally reported heat as more painful than men. Examiner gender had no main effect on verbal pain ratings [_F(1, 36)_ = 0.93, *p* = 0.34, ${\text{η}}_{\text{p}}^{2}$ = 0.03]. However, there was a significant interaction effect for participant and experimenter gender on verbal pain rating [*F*_(1,36)_ = 5.61, *p* = 0.02, ${\text{η}}_{\text{p}}^{2}$ = 0.14]. Bonferroni corrected *post-hoc* analysis revealed that female participants verbally reported higher tonic heat pain than males in the presence of a female examiner (*t* = 3.21, *p* = 0.01). Order of day of testing did not influence the gender effect [*F*_(1,36)_ = 0.01, *p* = 0.91]. For average CoVAS ratings, there were no significant main effects of participant [*F*_(1,36)_ = 1.20, *p* = 0.28, ${\text{η}}_{\text{p}}^{2}$ = 0.03] or examiner gender [*F*_(1,36)_ = 3.88, *p* = 0.06, ${\text{η}}_{\text{p}}^{2}$ = 0.10]. There was also no significant interaction effect [*F*_(1,36)_ = 2.70, *p* = 0.11, ${\text{η}}_{\text{p}}^{2}$ = 0.07] (Figure 2; Table 1). Order of session did not influence CoVAS ratings [*F*_(1,36)_ = 3.08, *p* = 0.09].

**Table 1:** Means and standard deviations and results of the repeated measures ANOVA tests, separated by male and female participants and examiners. ANOVA output for interaction effect presented.

	**Female examiner**		**Male examiner**		**RM-ANOVA^a^**
	**Mean (SD)**		**Mean (SD)**		
	**Females**	**Males**	**Females**	**Males**	***F*** (***p***)
PCT	45.36 (0.49)	45.48 (0.51)	45.45 (0.75)	45.70 (0.47)	0.45 (0.51)
CoVAS	29.83 (21.23)	40.34 (26.25)	28.45 (18.16)	27.50 (20.53)	2.70 (0.11)
Heat pain thresholds	43.64 (1.73)	43.5 (1.97)	43.84 (2.74)	44.93 (2.32)	1.59 (0.22)
Verbal rating	4.00 (2.03)	4.93 (2.25)	3.33 (1.64)	2.94 (1.52)	5.61 (0.02)

a *df = 19, interaction effect of participant gender x examiner gender*.

*RM-ANOVA, repeated measure ANOVA; PCT, participant controlled temperature; CoVAS, computerized visual analog scale*.

### Temperature Based Methods

There was no significant main effect of participant [heat pain thresholds: *F*_(1,36)_ = 1.80, *p* = 0.19, ${\text{η}}_{\text{p}}^{2}$ = 0.05; PCT: *F*_(1,36)_ = 1.02, *p* = 0.32, ${\text{η}}_{\text{p}}^{2}$ = 0.03] or examiner gender [heat pain thresholds: *F*_(1,36)_ = 2.64, *p* = 0.11, ${\text{η}}_{\text{p}}^{2}$ = 0.07; PCT: *F*_(1,36)_ = 3.31, *p* = 0.08, ${\text{η}}_{\text{p}}^{2}$ = 0.08]. There was also no significant interaction effect between participant and examiner gender [heat pain thresholds: *F*_(1,36)_ = 1.59, *p* = 0.22, ${\text{η}}_{\text{p}}^{2}$ = 0.04; PCT: *F*_(1,36)_ = 0.45, *p* = 0.51, ${\text{η}}_{\text{p}}^{2}$ = 0.01] (Figure 2; Table 1). Order of session did not influence PCT scores [*F*_(1,36)_ = 0.56, *p* = 0.46] or pain thresholds [*F*_(1,36)_ = 0.66, *p* = 0.42].

We also ran a repeat measures ANOVA on the initial rating of the PCT stimulus to investigate if there was a gender difference in this initial perception. There was no significant difference between genders [*F*_(1,36)_ = 2.417, *p* = 0.129], nor was there an effect of examiner gender [*F*_(1,36)_ = 1.490, *p* = 0.230]. Average initial rating for female participants was 5.55 ± 1.56 when tested by the male examiner and 5.80 ± 1.64 when tested by the female examiner. Average initial rating for male participants was 4.83 ± 1.58 when tested by the male examiner and 5.00 ± 1.57 when tested by the female examiner. Additionally, 33/38 participants reported the same pain rating at the beginning and end of the PCT test, 4 were within ±1/10 on an NRS, and 1 participant was within ±2/10 on an NRS. This is in contrast to the CoVAS test, where the range was ±3/10 on the NRS.

### PCS Correlations to Pain Outcomes

PCS subscales were not correlated to any pain outcomes in both males and females, and were also not correlated to relative difference in pain outcomes between examiners (Table 2).

**Table 2:** Correlations coefficients (R) between pain catastrophizing subscales and pain measurements adjusted for multiple comparisons (Bonferroni).

	**Male examiner**				**Female examiner**				**Difference scores between**			
									**male and female examiners**			
	**CoVAS**	**PCT**	**PT**	**Verbal**	**CoVAS**	**PCT**	**PT**	**Verbal**	**CoVAS**	**PCT**	**PT**	**Verbal**
Rumination	0.16	−0.16	−0.23	0.12	0.10	−0.23	−0.16	0.22	0.05	0.10	−0.05	−0.13
Magnification	0.14	−0.14	−0.21	0.01	0.09	−0.05	−0.11	0.08	0.05	−0.06	−0.08	−0.09
Helplessness	0.23	−0.32	−0.28	0.20	0.20	−0.03	−0.09	0.28	0.00	−0.22	−0.16	−0.13
Total	0.21	−0.24	−0.27	0.14	0.16	−0.11	−0.13	0.23	0.03	−0.08	−0.11	−0.13

## Discussion

The impact of examiner characteristics on study outcomes have been attributed a causal role in the ongoing scientific replication crisis (3). Among concerns is that the gender of the examiner contributes to heterogeneous outcomes between studies. As predicted by the Gender Context Model of Pain, we observed that sex differences in tonic heat pain perception may be exaggerated by verbal rating-based methods when the examiner is female. CoVAS pain ratings demonstrated similar trends, albeit not significant. In contrast, temperature-based methods of assessing heat pain were not significantly affected by the gender of the examiner.

To our knowledge, the effect of examiner gender on pain outcomes has been explicitly tested in six previous studies (see Table 3 for description) (9, 10, 17–19, 22). For subjective pain ratings, our observations correspond with those reporting an opposing examiner gender effect (9, 10, 17, 18) as well as social theories of pain which propose the gender context in which pain is expressed influences pain report (12). The former was evidenced in our reported verbal ratings in women, which were significantly higher in the presence of a female compared to a male examiner. Similar, albeit more variable results were observed for CoVAS ratings to heat pain. Our findings support the notion that pain communication may be more affected by gender interactions as compared to the actual pain experience. For example, when comparing verbal pain ratings to CoVAS ratings, the pain experience (CoVAS) was comparable, while the act of reporting to the experimenter verbally was influenced my experimenter gender. The notion that pain communication, but not experience, is influenced by gender is supported by a previous study that showed biological responses to pain (e.g., autonomic changes) are unaffected by examiner gender (17). Taken together, our findings provide evidence for a dissociation between pain experience and pain reporting, which is influenced by examiner gender. Overall, this lends support to the Gender Context Model of Pain (12), in that outcomes with the most social communication were more influenced by experimenter gender.

**Table 3:** Summary of studies examining the effect of examiner gender on pain outcomes.

**References**	**Test stimuli**	**Rating method**	**Gender effects**	**Additional measures**	**Study design**
Levine and De Simone (9)	**Cold pressor** Both hands in 0-1°C ice bucket	**Pain intensity** Numeric rating scale, given every 15 s for 180 s	**Intensity** Male participants reported lower pain intensity to a female experimenter	**Pain Affective scale** • Males reported less negative affective words to female experimenter	Parallel experimental design **Participants** *n* = 68 (33 female, 35 male) Ages 17-29 (M = 19.13)
Kallai et al. (18)	**Cold pressor** Non-dominant hand in circulating −1°C ice bucket	**Pain intensity** 10-point rating scale, given immediately after CPT **Pain threshold** Seconds **Pain tolerance** Seconds	**Intensity** Both male and female participants reported higher pain intensity to a female experimenter **Tolerance** Female participants had higher pain tolerances with a male experimenter Male participants had higher pain tolerances with a female experimenter **Threshold** No experimenter gender effect found for pain threshold	Participants rated the examiner's authority, competence, likeability and masculinity/femininity on seven-point rating scales	Parallel experimental design **Participants** *n* = 160 (80 female, 80 male) Female ages 17-36 (M = 23.19, SD 3.59) Male ages 19-59 (M = 24.55, SD 5.79)
Gijsbers and Nicholson (10)	**Pressure** Pressure algometer with 0-9 kg force range on upper sternum	**Pain threshold** kilograms	**Threshold** Male participants had higher pain thresholds with a female examiner	**Anxiety** • Measured with 10 cm VAS • Anxiety was low for both female and male participants • No correlation with pain thresholds **McGill Pain Questionnaire** • No significant examiner gender effect on pain scores • Indicated low emotional concern in participants	Parallel experimental design **Participants** *n* = 64 (32 females, 32 males) Female ages 18-36 (M = 21.0, SD 4.4) Male ages 18-49 (M = 23.0, SD 8.1)
Weisse et al. (19)	**Cold pressor** Non-dominant hand in 0-2°C ice bucket	**Pain intensity** 0–20 rating scale every 15 s for a total of 300 s	**Intensity** No main effect found for pain reporting and examiner gender. However, an interaction was found with participant race and examiner gender: Black participants reported higher pain intensities than white participants to a female examiner	**Pain unpleasantness scale** • No main effect for pain reporting and examiner gender • An interaction found for participant race and examiner gender: black participants reported more unpleasantness than white participants to a female examiner	Parallel experimental design **Participants** *n* = 343 (187 females, 156 males) Ages 17-43 (M = 20.27)
Aslaksen et al. (17)	**Heat** TSA II Neurosensory Analyzer (Medoc, Israel): 30 ∙ 30 mm aluminium contact thermode with a 10°C/s change rate on right forearm	**Pain intensity** 100 mm VAS **Physiological** **pain response** Heartrate variability and skin conductance levels	**Intensity** Male participants reported lower pain intensity to a female examiner Physiological pain response: No examiner gender effect found for physiological responses	**Pain unpleasantness scale** • No significant examiner gender effect • Short **Adjective Check List ** **and Self-Assessment** **Manikin scale** • Male participants reported lower arousal to female experimenters • No significant examiner gender effect with subjective stress or mood scales	Parallel experimental design **Participants** *n* = 64 (32 females, 32 males) Female ages 19-40 (M = 23.61, SD 3.99) Male ages 19-35 (M = 23.3, SD 2.49)
Vigil et al. (22)	**Cold pressor** One of two CPT protocols used on left hand: (1) 5°C ± 1°C circulating ice bucket, or (2) Isotemp 6200R28 (Fisher Scientific, USA) electromechanical CPT device at 5°C ± 0.1°C	**Pain intensity** 10-point VAS, 30 s into CPT **Pain threshold** Seconds **Pain tolerance** Seconds	**Intensity** Both male and female participants reported higher pain intensity to a female examiner **Tolerance** Subjects had higher pain tolerances with a male examiner **Threshold** No examiner gender effect found for pain threshold	No additional measures performed	Parallel experimental design **Participants** *n* = 352 (48% males) Ages 18-30 (M = 19.8, SD 2.1)

*CPT, cold pressor test*.

The modernization of QST assessments has seen a shift to temperature-based methods, including standardized methods of measuring heat pain thresholds (6). Previous studies exploring experimenter gender effects (Table 3) have not incorporated temperature-based methods of assessing pain, relying instead on verbal ratings or time-based approaches that assess tolerance (e.g., cold pressor). To address this limitation, we assessed examiner gender effects on pain threshold determined by method of limits and PCT. The latter, a revitalized approach based a method originally established by Hardy and Greene (23), involves participants continuously adjusting the temperature of the thermode over 2 min in order to maintain their initial perception of noxious heat (15). The concept of PCT is similar to CoVAS, but dynamic aspects of pain (i.e., the fluctuations in the perception of a constant painful stimuli over time) are reflected by changes in temperature as opposed to continuous ratings (15). Compared to CoVAS and verbal pain ratings, PCT provides pain reporting with the least obvious social context. Where verbal pain ratings involved direct communication with examiners and CoVAS involved the perceived communication of digital 0-10 scale, PCT involves button clicks to maintain sensation. To that end, PCT was more resilient to gender effects compared to verbal or CoVAS outcomes, as examiner gender did not significantly influence PCT. These findings provides further support for the social context of pain model, as PCT is less clearly a “rating” of pain to an examiner, and thus less influenced by the social context (12).

To consider a potential psychosocial factor, we aimed to explore the relationship between participants' PCS scores and variability introduced by the gender of the examiner. For pain catastrophizing, we observed no significant associations between PCS scores and any pain outcomes, for both raw scores as well as evaluating relative differences in pain outcomes between examiners. This suggests that pain catastrophizing does not have a significant influence on our observed gender effects on pain outcomes.

### Limitations

Our findings are limited to a relatively homogenous population (i.e., undergraduate and graduate students). The extent our results are generalizable to other populations (e.g., older, community dwelling adults) requires further study. We also did not collect or report relationships between the race, ethnicity, height, or weight of our participants or examiners and the possible effects on pain ratings. This was beyond the scope of our current study and represents another avenue for further exploration. To that end, we did not control for experimenter ethnicity, or other examiner characteristics (e.g., hair color, eye color etc.). We sought to maintain ecological validity in our selection of a male and female examiner, rather than overly constrain various aspects of personal appearance/characteristics. To that end, our findings are based on the effect of clearly male and clearly female examiners.

In comparison to previous investigations of experimenter gender effects on pain perception (9, 10, 17–19, 22), our study is limited to a relatively small sample size. However, as a seminal study to explore experimenter gender effects on multiple heat-pain outcomes, our sample size was chosen pragmatically and in accordance with a sample size calculation related to quantitative pain assessments previously used in similar experimenter gender comparisons. We were unable to collect data on two participants due to technical issues, resulting in a fewer number of participants than reported in our a priori power calculation. We reported ${\text{η}}_{\text{p}}^{2}$ values for all repeated measures ANOVA analyses along with Cohen's d values to highlight within experimenter effects. Finally, our findings are also limited to our included heat pain-based assessment methods. Future studies should continue to explore experimenter gender effects in other pain outcomes making use of differing modalities, such as mechanical pinpricks and more modern cold pain assessments.

We did not have our examiners conform to stereotypical gender roles, which may have muted examiner effects. Studies whose examiners dressed in “stereotypical gender conforming” ways (9, 10, 17, 18) appear more likely to see significant examiner effects compared to those that did not control for dress (24–31). Status of the examiner may also matter—participants of both genders report higher pain tolerance to “high status” (i.e., professionally dressed, used formal names) examiners (18). In the present study we attempted to control for gender stereotypes through recruiting peer examiners that wore a uniform—lab coat over pants and a t-shirt—and that used the same script. This moderate “de-gendering” of the examiners and reduction of potential power imbalances through using peers may have reduced gender differences in the heat pain assessments.

Also, our study and those previous have focused on participants that conformed to gender norms. It is not clear if those who do not conform to gender norms may report pain differently or have different examiner-participant gender interaction effects in the reporting of pain. Examining pain in transgendered and non-binary individuals represents an important and understudied area of pain science—an area that would not only shed light on a marginalized populations' pain experience, but would also extend our understanding of the interaction between gender and pain.

### Conclusions and Future Implications

Overall, our findings are aligned with the Gender Context Model of Pain, insofar as those outcome measures that were most likely to be influenced by social factors (i.e., verbal pain ratings) were more susceptible to experimenter gender effects, while outcomes less likely to be influenced by social factors (i.e., PCT) were not significantly influenced by experimenter gender. The examiner and participant gender can both influenced pain reporting, with the perceived level of examiner-participant interaction appearing to mediate these effects. Researchers should consider the social environment of their experiments, the pain measurement used, and the gender of their experimenters as these factors all play a role in detecting sex/gender differences in pain measurements. The use of non-verbal pain measures, with little to no examiner influence (e.g., coded temperature information *via* PCT) may be a potential solution to circumvent the effects of experimenter gender on pain related outcomes.

## Data Availability Statement

The raw data supporting the conclusions of this article will be made available by the authors, without unduereservation.

## Ethics Statement

The studies involving human participants were reviewed and approved by Behavioral Research Ethics Board at the University of British Columbia. The patients/participants provided their written informed consent to participate in this study.

## Author Contributions

JM, LL, and JK designed and directed the project. NB and RB collected the data and ran the statistics, with assistance and supervision by JM and LL. Figures were created by JM and LL. JM wrote the manuscript, with all authors contributing to the final version.

## Funding

JK was supported by a NSERC discovery grant.

## Conflict of Interest

The authors declare that the research was conducted in the absence of any commercial or financial relationships that could be construed as a potential conflict of interest.

## Publisher's Note

All claims expressed in this article are solely those of the authors and do not necessarily represent those of their affiliated organizations, or those of the publisher, the editors and the reviewers. Any product that may be evaluated in this article, or claim that may be made by its manufacturer, is not guaranteed or endorsed by the publisher.
